# Comparative effectiveness of urate lowering with febuxostat versus allopurinol in gout: analyses from large U.S. managed care cohort

**DOI:** 10.1186/s13075-015-0624-3

**Published:** 2015-05-12

**Authors:** Jasvinder A Singh, Kasem S Akhras, Aki Shiozawa

**Affiliations:** Medicine Service and Center for Surgical Medical Acute care Research and Transitions (C-SMART), Birmingham VA Medical Center, 700 South 19th Street, Birmingham, AL 35233 USA; Department of Medicine at School of Medicine, University of Alabama, 1670 University Boulevard, Birmingham, AL 35233 USA; Division of Epidemiology at School of Public Health, University of Alabama, 1665 University Boulevard, Birmingham, AL 35233 USA; Department of Orthopedic Surgery, Mayo Clinic College of Medicine, 200 1st St SW, Rochester, MN 55905 USA; Takeda Pharmaceuticals International, Inc., One Takeda Parkway, Deerfield, IL 60015 USA; University of Alabama, Faculty Office Tower 805B, 510 20th Street S, Birmingham, AL 35294 USA

**Keywords:** Gout, Comparative effectiveness, Serum urate, Febuxostat, Allopurinol

## Abstract

**Introduction:**

To assess the comparative effectiveness of febuxostat and allopurinol in reducing serum urate (sUA) levels in a real-world U.S. managed care setting.

**Methods:**

This retrospective study utilized 2009 to 2012 medical and pharmacy claims and laboratory data from a large U.S. commercial and Medicare Advantage health plan. Study patients had at least one medical claim with a diagnosis of gout, at least one filled prescription for febuxostat or allopurinol and at least one sUA measurement post-index prescription. Reduction in sUA was examined using propensity score-matched cohorts, matched on patient demographics (gender, age), baseline sUA, comorbidities, geographic region and insurance type.

**Results:**

The study sample included 2,015 patients taking febuxostat and 14,025 taking allopurinol. At baseline, febuxostat users had a higher Quan-Charlson comorbidity score (0.78 vs. 0.53; *P* <0.001), but similar age and gender distribution. Mean (standard deviation (SD)) sUA level following propensity score matching among treatment-naïve febuxostat vs. allopurinol users (n = 873 each) were: pre-index sUA, 8.86 (SD, 1.79) vs. 8.72 (SD, 1.63; *P* = 0.20); and post-index sUA, 6.53 (SD, 2.01) vs. 6.71 (SD, 1.70; *P* = 0.04), respectively. A higher proportion of febuxostat users attained sUA goals of <6.0 mg/dl (56.9% vs. 44.8%; *P* <0.001) and <5.0 mg/dl (35.5% vs. 19.2%; *P* <0.001), respectively. Time to achieve sUA goals of <6.0 mg/dl (346 vs. 397 days; *P* <0.001) and <5.0 mg/dl was shorter in febuxostat vs. allopurinol users (431 vs. 478 days; *P* <0.001), respectively. Similar observations were made for overall propensity score-matched cohorts that included both treatment-naïve and current users (n = 1,932 each).

**Conclusions:**

Febuxostat was more effective than allopurinol at the currently used doses (40 mg/day for febuxostat in 83% users and 300 mg/day or lower for allopurinol in 97% users) in lowering sUA in gout patients as demonstrated by post-index mean sUA level, the likelihood of and the time to achieving sUA goals.

**Electronic supplementary material:**

The online version of this article (doi:10.1186/s13075-015-0624-3) contains supplementary material, which is available to authorized users.

## Introduction

Gout is the most common inflammatory arthritis in adults, affecting 3.9% of the U.S. population [1]. The prevalence of gout far exceeds that of rheumatoid arthritis (RA) at 1% [2], the prototype of inflammatory arthritis in adults. Gout is associated with significant morbidity, functional limitation and health-related quality of life (HRQOL) deficits [3-5] as well as increased cardiovascular morbidity and mortality [6-9]. Optimal treatment of gout is based on two principles: adequate chronic use of urate-lowering therapies (ULT; a xanthine oxidase (XO) inhibitor and uricosurics) aiming to achieve target serum urate (sUA) levels and anti-inflammatory therapies for acute flares and anti-inflammatory prophylaxis [10]. Adequate lowering of sUA to a target level of <6.0 mg/dl is associated with lower risk of acute flares [11] and better function and quality of life [12], and is cost effective in various health care settings [13-17]. Thus, achievement of target sUA <6.0 mg/dl is key to quality management of gout [18-20]. With 8.3 million U.S. adults suffering from gout [1], appropriate sUA lowering will likely reduce its public health burden and associated cost.

Comparative effectiveness research (CER) is a high priority area for research, practice and policy-making and recent commitment of $1.1 billion to CER by the American Recovery and Reinvestment Act of 2009 underscores its importance to health policy [21]. For 50 years, a single purine XO inhibitor, allopurinol, was available in the U.S. [22,23]. Since allopurinol is available as a generic medication and is an effective ULT, it is used in >95% of cases, while uricosurics are used infrequently for the treatment of hyperuricemia in gout [24-26]. In 2009, a non-purine XO inhibitor, febuxostat, was approved by the U.S. Food and Drug Administration for the treatment of gout [27]. We aimed to assess the patterns of use of allopurinol and febuxostat in a large managed care organization (MCO) and perform a CER study comparing the ability of allopurinol and febuxostat to lower sUA. Our main study objective was to study the change in sUA with allopurinol versus febuxostat treatment by assessing the proportion of patients achieving a post-index sUA goal of <6.0 mg/dL in the follow-up period, a clinically meaningful and important outcome for patients with gout [11-16]. As a secondary objective, we compared serum creatinine (SCr) levels between allopurinol and febuxostat users.

## Methods

### Setting, participants and data sources

Methods and results are described as recommended in the STrengthening of Reporting in OBservational studies in Epidemiology (STROBE) statement [28]. This retrospective study analyzed U.S. patients with a prescription for either febuxostat or allopurinol for the treatment of gout from February 1, 2009 to May 31, 2012. Medical data, pharmacy data, enrollment information and laboratory results from study subjects from both Medicare Advantage plans and a commercial plan were obtained from the Optum Research Database (ORD). Patients in this dataset are similar to U.S. insured population in terms of race, gender, age, and geographic distribution, which has been described previously [29]. All administrative claims data were de-identified and study procedures adhered to the provisions of the Health Insurance Portability and Accountability Act (HIPAA) of 1996. Because this study did not involve the collection, use, or transmittal of individually identifiable data, Institutional Review Board review or approval was not required and no patient consent was needed.

Commercial and Medicare Advantage health plan enrollees were identified between February 1, 2009 and May 31, 2012 (the identification period). Medicare Advantage is a managed Medicare health insurance plan offered by private insurers [30]. To be eligible for study inclusion, patients must have met both of the following criteria during the identification period: 1) had at least one medical claim with an International Classification of Diseases, ninth revision (ICD-9) diagnosis code for gout (274.xx); and 2) had at least one pharmacy claim for allopurinol or febuxostat. This time frame included the febuxostat launch date. The date of the first febuxostat or allopurinol filled prescription was defined as the patient’s index date; if a patient had a filled prescription for both febuxostat and allopurinol, then the first filled prescription for febuxostat was defined as the index date. Patients were excluded if they were younger than 18 years as of the year of index date; had evidence of cancer or rheumatoid arthritis during the study period; had no sUA laboratory result 14 or more days after the index date (the primary outcome measure); had less than six months of continuous enrollment prior to their index date (baseline period); or had <90 days follow-up after index prescription of allopurinol or febuxostat. In order to control for confounding, baseline data were obtained during the six-month baseline period prior to the index date. Patients were followed until August 30, 2012 or until the patient was no longer enrolled in the health plan, whichever was earlier.

### Independent variable (drug exposure)

Patients were assigned to one of two study cohorts based on whether their index medication filled prescription was for febuxostat or allopurinol. Because febuxostat was approved in 2009, patients who received allopurinol and then switched to febuxostat were assigned to the febuxostat cohort. Patients were not excluded from the study if they had evidence of febuxostat or allopurinol use during the baseline period. Allopurinol-treated patients were allowed to have an index medication dose between 100 and 1,500 mg/day, because allopurinol is available in various tablet strengths. Febuxostat-treated patients were included if they had an index medication dose 40 mg or 80 mg, because febuxostat is only available in these recommended doses. Patient demographics (age, gender and region) and baseline clinical characteristics (utilization and comorbid conditions assessed by Quan-Charlson comorbidity score [31]) were assessed. Gout is frequently associated with comorbidities [32-36].

### Study outcome measures

Outcomes were assessed during a variable follow-up period of at least three months following the index date. The main outcome measure was post-index mean sUA levels. The proportions of patients who achieved a target sUA <6 mg/dl or <5 mg/dl were also assessed. When patients had more than one post-index sUA level, the earliest value that attained goal was selected.

### Bias

We anticipated selection bias, that is, confounding by indication, because patient characteristics impact the choice of allopurinol vs. febuxostat. Therefore, we used propensity score matching (PSM) to minimize this bias. In the absence of chart review, some misclassification error due to use of codes for gout may have occurred. We did not think that this led to biased estimates, since there is no evidence that this may have occurred more often with one medication versus the other.

### Sample size

No formal sample size calculations were done *a priori*. All available patients who met the study inclusion and exclusion criteria were included in this analysis.

### Patient matching and statistical analysis

We used PSM methodology to account for selection/channeling bias [37]. Propensity scores were estimated by unconditional logistic regression analyses that incorporate predictors of therapy as independent variables in the regression and treatment cohort as the outcome. The propensity score was the fitted value of the probability of being a member of the febuxostat cohort given membership in the study population and the covariate pattern. To the extent that the clinical decision to use febuxostat in a particular patient depends on the health characteristics of the patient at the time of the decision, the propensity score modeled the clinical decision-making process. The covariates used in the propensity analysis included: age, gender, insurance type, region, baseline medication use (allopurinol, uricosurics), baseline comorbidities (kidney failure, kidney stones, dialysis, angina, diabetes, coronary artery disease, heart failure, myocardial infarction, stroke, peripheral artery disease, osteoarthritis, hypertension, hyperlipidemia, and gout flares), baseline sUA, baseline health care costs and the follow-up duration (post-index prescription period). For each febuxostat patient, an allopurinol patient with the closest propensity score (±0.01 units) was selected. Patients who were not matched were excluded from analysis. Febuxostat and allopurinol patients were matched in a 1:1 ratio. Following the propensity score match, all categorical variables were examined descriptively. Comparisons between the febuxostat and allopurinol cohorts for categorical outcomes of interest (post-index sUA goal <6.0 mg/dl and <5.0 mg/dl) were done using a McNemar’s test while continuous measures (pre- and post-index prescription sUA and SCr, time to sUA) were examined using a paired Student’s *t* test; an *a priori* two-tailed level of significance was set at the 0.05 level.

## Results

### Demographic and clinical characteristics

**Figure 1:**
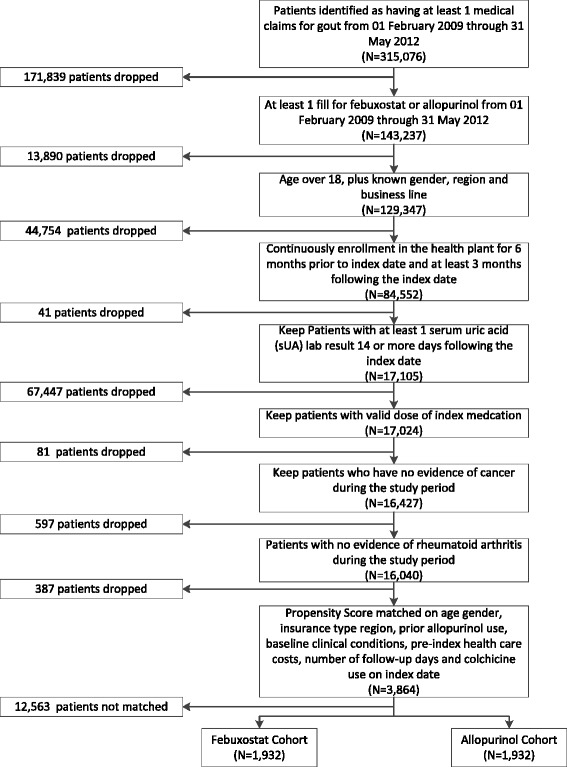
Each box represents a drop step, the N in the reach box represents the number of patients left following each drop step.

Nearly 315,076 commercial and Medicare Advantage patients had a medical claim indicating gout, and 143,237 of these patients also had a pharmacy claim for febuxostat or allopurinol. After application of continuous enrollment, laboratory result, dose and cancer criteria, a final sample of 16,040 patients was available for analysis (Figure 1). Of these, 2,015 patients were assigned to the febuxostat cohort and 14,025 were assigned to the allopurinol cohort. Table 1 presents characteristics of the study sample before and after matching. The post-index follow-up periods in these unmatched patient populations differed somewhat and were as follows: allopurinol group, 224 days (standard deviation (SD), 256); and febuxostat group, 188 days (SD, 220). Therefore, the cohorts were matched on the post-index follow-up duration, among other variables, to avoid selection bias. More than 80% of patients were male in both cohorts.

**Table 1:** **Patient demographics and clinical characteristics pre- and post-matching**

	**Pre-matched**				**Fully matched**				**Treatment-naïve matched**			
	**Total (N = 16,040)**	**Febuxostat (N = 2,015)**	**Allopurinol (N = 14,025)**	***P*** **value** ^**a**^	**Total (N = 3,864)**	**Febuxostat (N = 1,932)**	**Allopurinol (N = 1,932)**	***P*** **value** ^**a**^	**Total (N = 1,746)**	**Febuxostat (N = 873)**	**Allopurinol (N = 873)**	***P*** **value** ^**a**^
Age, **mean (SD)**	55.9 (12.0)	56.0 (12.3)	55.9 (12.0)	0.81	55.6 (12.2)	56.0 (12.3)	55.2 (12.2)	0.076	55.4 (12.3)	55.2 (12.0)	55.6 (12.7)	0.57
**Quan-Charlson comorbidity score, mean (SD)**	0.56 (1.12)	0.78 (1.35)	0.53 (1.07)	<0.001	0.68 (1.23)	0.75 (1.30)	0.61 (1.16)	<0.001	0.64 (1.16)	0.69 (1.22)	0.58 (1.10)	0.063
	**n (%)**	**n (%)**	**n (%)**		**n (%)**	**n (%)**	**n (%)**	***P*** **value** ^**a**^	**n (%)**	**n (%)**	**n (%)**	***P*** **value** ^**a**^
**Age**												
18-44	2,856 (17.8%)	388 (19.3%)	2,468 (17.6%)	0.07	742 (19.2%)	367 (19.0%)	375 (19.4%)	0.74	348 (19.9)	178 (20.4)	170 (19.7)	0.63
45-64	9,402 (58.6%)	1,123 (55.7%)	8,279 (59.0%)	0.005	2,196 (56.8%)	1,086 (56.2%)	1,110 (57.5%)	0.44	974 (55.8)	506 (58.0)	468 (53.6)	0.07
65-74	2,660 (16.6%)	356 (17.7%)	2,304 (16.4%)	0.16	661 (17.1%)	339 (17.6%)	322 (16.7%)	0.47	298 (17.1)	131 (15.0)	167 (19.1)	0.02
75 and older	1,122 (7.0%)	148 (7.3%)	974 (6.9%)	0.51	265 (6.9%)	140 (7.3%)	125 (6.5%)	0.34	126 (7.2)	58 (6.6)	68 (7.8)	0.36
**Gender**												
Male	13,607 (84.8%)	1,682 (83.5%)	11,925 (85.0%)	0.07	3,247 (84.0%)	1,610 (83.3%)	1,637 (84.7%)	0.24	1,457 (83.4)	740 (84.8)	717 (82.1)	0.14
Female	2,433 (15.2%)	333 (16.5%)	2,100 (15.0%)		617 (16.0%)	322 (16.7%)	295 (15.3%)		289 (16.5)	133 (15.2)	156 (17.9)	0.14
**Insurance type**				0.04				0.33				<0.001
Commercial	13,429 (83.7%)	1,656 (82.2%)	11,773 (83.9%)		3,203 (82.9%)	1,590 (82.3%)	1,613 (83.5%)		1,433 (82.1)	746 (85.4)	687	
Medicare Advantage	2,611 (16.3%)	359 (17.8%)	2,252 (16.1%)		661 (17.1%)	342 (17.7%)	319 (16.5%)		313 (17.9)	127 (14.5)	186	
**Geographic location**												
Northeast	1,168 (7.3%)	137 (6.8%)	1,031 (7.4%)	0.37	280 (7.3%)	132 (6.8%)	148 (7.7%)	0.32	137 (7.8)	74 (8.5)	63 (7.2)	0.33
Midwest	1,450 (9.0%)	143 (7.1%)	1,307 (9.3%)	0.001	273 (7.1%)	140 (7.3%)	133 (6.9%)	0.66	111 (6.4)	49 (5.6)	62 (7.1)	0.20
South	11,020 (68.7%)	1,482 (73.6%)	9,538 (68.0%)	<0.001	2,811 (72.8%)	1,415 (73.2%)	1,396 (72.3%)	0.49	1,256 (71.9)	640 (73.3)	616 (70.6)	0.20
West	2,402 (15.0%)	253 (12.6%)	2,149 (15.3%)	0.001	500 (12.9%)	245 (12.7%)	255 (13.2%)	0.63	242 (13.9)	110 (12.6)	132 (15.1)	0.13
**Allopurinol use**	5,543 (34.6%)	748 (37.1%)	4,795 (34.2%)	0.010	1,306 (33.8%)	679 (35.1%)	627 (32.5%)	0.08				
**Comorbidities**												
Kidney failure	310 (1.9%)	77 (3.8%)	233 (1.7%)	<0.001	126 (3.3%)	61 (3.2%)	65 (3.4%)	0.72	43 (2.5%)	23 (2.6%)	20 (2.3%)	0.64
Heart failure	772 (4.8%)	141 (7.0%)	631 (4.5%)	<0.001	256 (6.6%)	126 (6.5%)	130 (6.7%)	0.80	106 (6.1%)	55 (6.3%)	51 (5.8%)	0.69
Peripheral arterial disease	200 (1.3%)	34 (1.7%)	166 (1.2%)	0.06	65 (1.7%)	30 (1.6%)	35 (1.8%)	0.53	24 (1.4%)	10 (1.1%)	14 (1.6%)	0.64
Osteoarthritis	2,686 (16.8%)	418 (20.7%)	2,268 (16.2%)	<0.001	760 (19.7%)	388 (20.1%)	372 (19.3%)	0.52	325 (18.6%)	155 (17.7%)	170 (19.5%	0.36
Hypertension	9,844 (61.4%)	1,368 (67.9%)	8,476 (60.4%)	<0.001	2,598 (67.2%)	1,297 (67.1%)	1,301 (67.3%)	0.89	1,145 (65.6%)	572 (65.5%)	573 (65.6%)	0.96
Hyperlipidemia	8,857 (55.2%)	1,161 (57.6%)	7,696 (54.9%)	0.021	2,221 (57.5%)	1,114 (57.7%)	1,107 (57.3%)	0.82	984 (56.4%)	489 (56.0%)	495 (56.7%)	0.77
	**Mean (SD)**	**Mean (SD)**	**Mean (SD)**	***P*** **value**	**Mean (SD)**	**Mean (SD)**	**Mean (SD)**	***P*** **value**	**Mean (SD)**	**Mean (SD)**	**Mean (SD)**	***P*** **value**
Baseline sUA	8.3 (2.0)	8.4 (2.2)	8.2 (2.0)	0.036	8.3 (2.1)	8.4 (2.2)	8.3 (2.0)	0.97	8.7 (1.8)	8.7 (2.0)	8.7 (1.7)	0.85

*P value is for comparison of febuxostat and allopurinol. SD, standard deviation; sUA, serum urate.

In the pre-matched analysis, patients taking febuxostat, compared to those taking allopurinol, were more likely to be covered by a Medicare Advantage plan (17.8% vs. 16.1%; *P* = 0.045); less likely to be living in the Midwest (7.1% vs. 9.3%, *P* = 0.001) or West (12.6% vs. 15.3%, *P* = 0.001); and more likely to be residing in the South (73.6% vs. 68.0%, *P* <0.001) (Table 1). In the pre-matched analysis, compared to allopurinol-treated patients, febuxostat-treated patients had: higher proportion with pharmacy claims for uricosuric medication (1.8% vs. 0.9% for probenecid prescription; *P* <0.001, data not shown); significantly higher rates of kidney failure, heart failure, osteoarthritis and hypertension (*P* <0.001 each), and hyperlipidemia (*P* = 0.02; Table 1); and higher mean Quan-Charlson comorbidity score (0.78 vs. 0.53, respectively; *P* <0.001). Fewer than 0.5% of subjects in the allopurinol cohort had a daily dose greater than 800 mg/day.

Twenty-four percent of febuxostat patients had switched from allopurinol. Mean (SD) allopurinol and febuxostat doses prior to sUA target <6 mg/dl were as follows: 284 mg/day (SD, 123 mg/day) and 54 mg/day (SD, 22 mg/day). Following PSM, no significant differences were observed in demographic characteristics, or frequencies of comorbidities (Table 1).

### Main analysis: serum urate (sUA) in treatment-naïve subjects

There were 873 matched pairs that had no evidence of treatment with allopurinol or febuxostat in the pre-index period (Table 2). The most common doses were 300 mg/day or lower dose for allopurinol and 40 mg/day for febuxostat (Table 2). Patterns of use of anti-inflammatory prophylaxis and index prescription dose change are shown in Table 2. Rates of comorbidities during the post-index period were similar between cohorts in the propensity score-matched analysis (Table 3).

**Table 2:** **Patient treatment patterns among the treatment-naïve propensity score-matched study population (N = 1,746)**

**Post-index treatment patterns**	**Total**		**Febuxostat**		**Allopurinol**		**Febuxostat vs. allopurinol**
	**(N = 1,746)**		**(N = 873)**		**(N = 873)**		***P*** **value**
	**N**	**%**	**n**	**%**	**n**	**%**	
**Prophylaxis treatment**	940	53.8%	510	58.4%	430	49.3%	<0.001
Steroids	597	34.2%	323	37.0%	274	31.4%	0.013
NSAIDs	362	20.7%	180	20.6%	182	20.9%	0.906
Colchicines	354	20.3%	225	25.8%	129	14.8%	<0.001
**Index febuxostat dose**							
40 mg	726	41.6%	726	83.2%	-	-	-
80 mg	147	8.4%	147	16.8%	-	-	-
**Index allopurinol dose**							
100 mg	350	20.1%	-	-	350	40.1%	-
101-299 mg	129	7.4%	-	-	129	14.8%	-
300 mg	369	21.1%	-	-	369	42.3%	-
>300 mg	25	1.4%	-	-	25	2.9%	-
**Index medication dose change**	458	26.2%	160	18.3%	298	34.1%	<0.001
**Percent of dose change**							
51-99% reduction	28	1.6%	0	0.0%	28	3.2%	<0.001
1-50% reduction	38	2.2%	6	0.7%	32	3.7%	<0.001
1-50% increase	46	2.6%	1	0.1%	45	5.2%	<0.001
51-100% increase	241	13.8%	143	16.4%	98	11.2%	0.002
>100% increase	105	6.0%	10	1.2%	95	10.9%	<0.001
	**Mean**	**SD**	**Mean**	**SD**	**Mean**	**SD**	
**Time to dose change (days)**	198	197	211	188	192	202	0.32
**Index medication dose immediately prior to sUA goal attainment of <6 mg/dl**	N/A	N/A	54.1	20.5	275.8	109.5	N/A
**Index medication dose immediately prior to sUA goal attainment of <5 mg/dl**	N/A	N/A	55.0	21.5	302.0	125.8	N/A

N/A, not applicable, since the dose for febuxostat and allopurinol are different and averaging of doses is meaningless. NSAIDs, nonsteroidal anti-inflammatory drugs; SD, standard deviation; sUA, serum urate.

**Table 3:** **Post-index clinical characteristics in the treatment-naïve propensity score-matched cohorts - (rates per 1,000 patient-years)**

	**Total**	**Febuxostat**	**Allopurinol**	**Allopurinol vs. febuxostat**
	**(N = 1,746)**	**(N = 873)**	**(N = 873)**	**Incidence rate ratio**
				**(95% confidence interval)**
**Kidney failure**	14.8	24.2	5.3	0.22 (0.08, 0.51)
**Kidney stones**	4.0	3.6	4.5	1.26 (0.32, 5.21)
**Dialysis**	7.4	7.9	6.8	0.85 (0.31, 2.27)
**Angina**	21.4	19.8	23.0	1.16 (0.67, 2.03)
**Diabetes**	76.0	85.6	66.4	0.78 (0.56, 1.08)
**Coronary artery disease**	80.1	75.1	85.3	1.13 (0.84, 1.53)
**Heart failure**	25.2	31.3	18.8	0.60 (0.35, 1.02)
**Myocardial infarction**	17.9	18.3	17.5	0.95 (0.52, 1.75)
**Stroke**	32.5	35.9	28.9	0.81 (0.51, 1.26)
**Peripheral arterial disease**	18.8	21.4	16.0	0.75 (0.40, 1.36)
**Osteoarthritis**	170.6	182.5	158.6	0.87 (0.70, 1.08)
**Hypertension**	231.0	234.4	227.5	0.97 (0.74, 1.27)
**Hyperlipidemia**	355.6	358.5	352.8	0.98 (0.80, 1.20)
**Alcohol abuse**	8.1	7.2	9.1	1.25 (0.50, 3.24)

**Figure 2:**
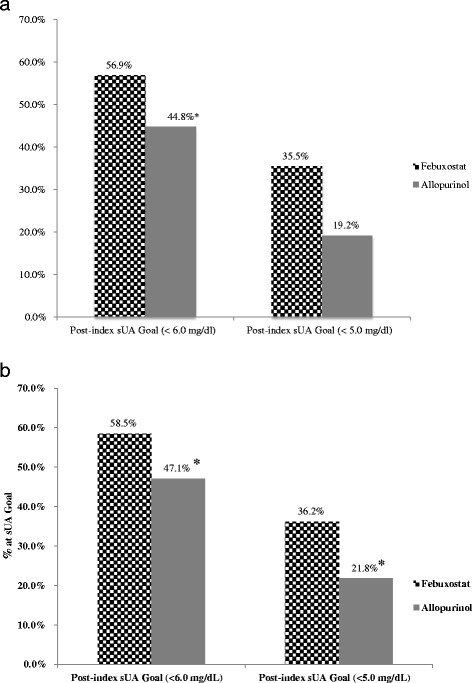
Y-axis represents the proportion that achieved target serum urate (sUA) in each group, febuxostat versus allopurinol. **a** provides the comparison for treatment-naive new users and **b** includes both treatment-naive or not-naive populations (i.e., new and current users).

In this subset, febuxostat patients were more likely to have commercial insurance vs. Medicare Advantage (85.5% vs. 78.7%, *P* <0.001). There were no differences between the febuxostat and allopurinol populations in baseline comorbidities or the overall comorbidity score. The final mean (SD) allopurinol and febuxostat doses prior to the target sUA achievement of <6 mg/dl were as follows: 276 mg/day (SD, 109 mg/day) and 54 mg/day (SD, 20 mg/day). Of the treatment-naïve febuxostat patients, 56.9% attained the sUA goal of <6.0 mg/dl compared to 44.8% of the allopurinol patients (*P* <0.001; Figure 2a). A total of 35.5% of the treatment-naïve febuxostat patients attained the sUA goal of <5.0 mg/dl versus 19.2% of the allopurinol patients (*P* <0.001; Figure 2a).

### Sensitivity analyses: propensity score-matched full study cohort, new and current users

Table 4 presents sUA and SCr results in the full propensity score-matched sample of patients, including new and current users (n = 1,932 matched pairs). Pre-index period mean sUA was non-significantly higher among febuxostat users, 8.52 in febuxostat users and 8.36 in allopurinol users (*P* = 0.29). The average post-index sUA was lower in febuxostat compared to the allopurinol users (6.41 vs. 6.64, *P* <0.001), a difference that is statistically, though perhaps not clinically, significant (Table 4). Index allopurinol dose and changes, prophylaxis treatments were similar to the treatment-naïve cohorts (Additional file 1). The pattern of post-index comorbidities was similar between propensity score-matched cohorts (Additional file 2).

**Table 4:** **Serum urate (sUA) and serum creatinine (SCr) results - febuxostat vs. allopurinol- overall propensity-matched population (n = 3,864)**

**sUA/SCr laboratory results**	**Total**		**Febuxostat**		**Allopurinol**		**Febuxostat vs. allopurinol**
	**(N = 3,864)**		**(N = 1,932)**		**(N = 1,932)**		***P*** **value**
	**N (%)**	**Mean (SD)**	**N (%)**	**Mean (SD)**	**N (%)**	**Mean (SD)**	
**Pre-index sUA result**	2,137 (55.3)		1,082 (56.0)		1,055 (54.6)		0.38
Number of sUA results		1.3 (0.7)		1.4 (0.8)		1.2 (0.5)	<0.001
Final sUA result		8.3 (2.1)		8.4 (2.2)		8.3 (2.0)	0.70
Average sUA result		8.4 (1.9)		8.5 (1.9)		8.4 (1.9)	0.06
**Pre-index SCr result**	2,298 (59.5)		1,151 (59.6)		1,147 (59.4)		0.90
Number of SCr results		1.7 (1.3)		1.7 (1.2)		1.6 (1.4)	0.02
Final SCr result		1.3 (0.6)		1.4 (0.6)		1.3 (0.6)	0.003
Average SCr result		1.3 (0.6)		1.4 (0.6)		1.3 (0.6)	0.001
**Post-index sUA result**	3,864 (100)		1,932 (100)		1,932 (100)		–
Number of sUA results		2.0 (1.6)		2.1 (1.7)		1.8 (1.5)	<0.001
Average sUA result		6.5 (1.8)		6.4 (2.0)		6.6 (1.7)	<0.001
**Post-index SCr result**	3,492 (90.4)		1,747 (90.4)		1,745 (90.3)		0.91
Number of SCr results		3.1 (3.3)		3.2 (3.1)		3.1 (3.4)	0.16
Average SCr result		1.3 (0.6)		1.3 (0.6)		1.2 (0.6)	<0.001
Proportion with and the time to target sUA achievement							
**Post-index sUA goal (<6.0 mg/dL)**	2,039 (52.8)		1,130 (58.5)		909 (47.1)		<0.001
**Time to sUA goal <6.0 mg/dL (days)**		379 (339)		348 (319)		410 (355)	<0.001
**Post-index sUA goal (<5.0 mg/dL)**	1,120 (29.0)		699 (36.2)		421 (21.8)		<0.001
**Time to sUA goal <5.0 mg/dL (days)**		472 (354)		443 (332)		501 (372)	<0.001

SD, standard deviation.

**Figure 3:**
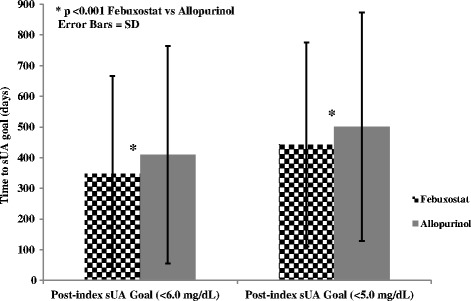
Y-axis represents the time to achieving target serum urate (sUA) in each group, febuxostat versus allopurinol. Error bars represent standard deviation.

Post-index target sUA goal attainment differed significantly between the cohorts (Figure 2b). A higher proportion of febuxostat users compared with allopurinol users attained the sUA goal of <6.0 mg/dL (58.5% vs. 47.1%, *P* <0.001) and the sUA goal <5.0 mg/dL (36.2% vs. 21.8%, *P* <0.001), findings very similar to those from the treatment-naïve population (Table 4; Figure 2b). Febuxostat users also had a shorter average length of time to target sUA goal attainment than allopurinol users (goal of <6.0 mg/dL: 348 days vs. 410 days, *P* <0.001; goal of <5.0 mg/dL: 443 days vs. 501 days, *P* <0.001) (Figure 3).

In the pre-index period, the final SCr result was 1.36 mg/dl among febuxostat users, and 1.28 mg/dl among allopurinol users (*P* <0.001) (Table 4). In the post-index period, the average SCr result was 1.31 mg/dl in febuxostat users, and 1.21 mg/dl in allopurinol users (*P* <0.001). The change from pre-index to mean post-index SCr result (a decrease in both cohorts) did not differ across treatment cohorts (−0.02 mg/dl in both cohorts (*P* = 0.82)).

## Discussion

In this study we found that in most commonly used doses, a significantly higher proportion of patients receiving febuxostat (most common dose, 40 mg/day) achieved a target sUA of <6.0 mg/dl and <5.0 mg/dl compared to those receiving allopurinol (most common dose, 300 mg/day or lower). Until recently, the treatment of hyperuricemia in gout mostly hinged on the appropriate use of a single drug, that is, allopurinol, since the use of uricosurics is uncommon. Since the U.S. launch of febuxostat in 2009, patients now have two XO inhibitors to choose from, allopurinol and febuxostat. Therefore, a comparative study of these two drugs is needed to help patients, providers and policy makers in making treatment decisions. These findings support the results observed in the pivotal clinical trials for febuxostat [33,38]. The target goals of serum urate <6.0 mg/dl and <5.0 mg/dl were achieved a month sooner in febuxostat-treated compared to allopurinol-treated patients. Several findings from this study deserve further discussion.

After matching patients on baseline characteristics, a significantly higher proportion of patients taking febuxostat than allopurinol achieved the target sUA level at commonly prescribed doses, with approximately 66% more patients achieving sUA <5.0 mg/dl (36.2% vs. 21.8%; relative difference) and a quarter more achieving sUA <6.0 mg/dl (58.5% vs. 47.1%). This difference is not only statistically significant, but also clinically meaningful. Patients receiving febuxostat (mostly at doses of 40 mg/day; 19% received 80 mg/day) achieved target sUA in one month less time than allopurinol-treated (most common dose 300 mg daily or lower; 5% received >300 mg/day) patients, a significant difference, both statistically and clinically. However, one must remember that in this effectiveness study, these measurements were not done at the pre-defined time, but at a time clinically indicated and as a part of routine clinical care. It may have taken a shorter time to achieve these target sUA levels.

These are important findings and indicate that febuxostat is an effective option for treatment of hyperuricemia in patients with gout. The higher rate of sUA testing in the febuxostat vs. allopurinol group (2.15 vs. 1.85 times, respectively) and a higher proportion of febuxostat-treated than allopurinol-treated patients getting a higher dose (19% received febuxostat 80 mg/day vs. 5% received allopurinol >300 mg/day), may have contributed to the ability to achieve target sUA, but it is unclear as to how much this contributed to the success rates. This needs to be examined in future studies. Our study extends similar findings by Kim *et al*. [39] in their unadjusted comparison of post-index sUA <6 mg/dl to a propensity score-adjusted analysis. Our study also adds new knowledge regarding both sUA goals and the time to achievement of both sUA goals of <6 and <5 mg/dl. Less than one-third of patients had a dose change in allopurinol and less than one-fifth in febuxostat doses after the index prescription, indicating that the dose titration as recommended by the guidelines to achieve target sUA is not a common practice [20].

These data from MCO enrollees reflect the common practices regarding treatment of hyperuricemia prevalent in the U.S. Just under half of patients using allopurinol received 300 mg/day (45%) with 50% getting <300 mg/day and only 5% getting >300 mg daily dose, as previously reported [24-26], and recently confirmed by Kim *et al*. [39] The demographics of patients in this dataset are similar to the U.S. commercially insured population, with similarities in age distribution and comorbidities to another gout study [39]. For example, 50% of individuals in this database were male, 73% were white, 8% were African American, and 9% were Hispanic. This database has previously been used to study ULT in gout patients [29]. While a higher dose of allopurinol is needed in many gout patients with higher body mass index, the most prescribed allopurinol dose for gout is still ≤300 mg daily [24-26]. Thus, the CER presented provides evidence for comparison of these most commonly used doses of allopurinol, not all allopurinol doses. It is likely that allopurinol dose of 300 mg/day is subtherapeutic in many patients with gout; lower allopurinol doses <300 mg/day may be even more likely to be subtherapeutic. There is an emerging trend to increase allopurinol dose to 800 to 1,500 mg/day until target sUA is achieved.

SCr decreased in both allopurinol and febuxostat users after treatment. This is an interesting finding, since gout is a metabolic disease with an effect on renal function. ULTs have been hypothesized to improve renal function, but definitive proof is lacking. A multicenter randomized trial assessing the impact of allopurinol on improving renal function in patients with diabetes with normal or moderately impaired kidney function was recently funded by the National Institutes of Health to answer this question more definitively [40]. Our observational study generates this hypothesis that can be tested in future randomized trials.

Our findings must be interpreted considering study limitations. Our study was observational, making it liable to residual confounding. Another limitation is that of selection bias, due to clinicians’ likelihood of choosing one or the other medication based on patient and/or disease characteristics. For example, compared to allopurinol users, febuxostat users may be expected to have had more severe gout and a higher rate of renal failure, since febuxostat was shown to be effective and safe in patients with renal failure. PSM was done to overcome selection bias. Misclassification error is possible, since we used ICD-9 codes to identify our study cohort. However, in a previous validation study at a Veterans Affairs Medical Center, 78% of patients with a database code for gout had evidence of this diagnosis in medical charts [41]. This was a real-world, observational analysis, and patient compliance with gout medication treatment may have varied over time. This may be one of the reasons that time to target sUA levels were more than one year on average in this analysis, a longer time period than is noted in clinical trials with controlled dosing environments. Quan-Charlson index score, our measure of comorbidity (renal disease, cardiac disease and so on), is a standard validated measure [31], but depends on the presence of ICD-9 codes in medical records, which raises the possibility of misclassification. We were unable to assess comparative safety of the two medications, due to limited resources. A small proportion of patients (5%) receiving allopurinol dose >300 mg/day of allopurinol limited us from comparing higher doses of allopurinol to febuxostat. Another study limitation is that we performed PSM only at baseline that included the duration of the medication exposure. The use of propensity scores at multiple follow-up times may allow for adjustment for other confounders; however, this could not be undertaken due to resource constraints.

## Conclusions

We found in this study that at the currently used doses, febuxostat (most common dose of 40 mg/day) was more effective in achieving the target sUA than allopurinol (most common doses of 300 mg/day or lower). The time to achieve target sUA in the febuxostat group is a month shorter than in the group receiving allopurinol. Slight improvements in renal function were noted with both allopurinol and febuxostat. These findings can inform patients and physicians when they are making a choice regarding the treatment of hyperuricemia. Obviously, the cost differences between the two treatments (febuxostat with much higher cost than allopurinol) should be taken into account. It is likely that individualized patient-physician decision-making that incorporates these data along with the risk of medication side effects and costs will lead to a more informed decision and a more satisfied patient. Future research with this data source will focus on the impact that the higher comparative effectiveness of febuxostat might have on health care costs.

## Additional files

Additional file 1:
**Patient treatment patterns among full matched study population (N = 3,864).**
Additional file 2:
**Post-index clinical characteristics in the propensity score-matched cohorts (%).**

## Abbreviations

CER: comparative effectiveness research
HRQOL: health-related quality of life
ICD-9: International Classification of Diseases, ninth revision
MCO: managed care organization
NSAIDs: nonsteroidal anti-inflammatory drugs
PSM: propensity score matching
SCr: serum creatinine
SD: standard deviation
sUA: serum urate
ULT: urate-lowering therapies
XO: xanthine oxidase
