# Erratum to: Comparative effectiveness of urate lowering with febuxostat versus allopurinol in gout: analyses from large U.S. managed care cohort

**DOI:** 10.1186/s13075-015-0752-9

**Published:** 2015-09-02

**Authors:** Jasvinder A. Singh, Kasem S. Akhras, Aki Shiozawa

**Affiliations:** Medicine Service and Center for Surgical Medical Acute care Research and Transitions (C-SMART), Birmingham VA Medical Center, 700 South 19th Street, Birmingham, 35233 AL USA; Department of Medicine at School of Medicine, University of Alabama, 1670 University Boulevard, Birmingham, 35233 AL USA; Division of Epidemiology at School of Public Health, University of Alabama, 1665 University Boulevard, Birmingham, 35233 AL USA; Department of Orthopedic Surgery, Mayo Clinic College of Medicine, 200 1st St SW, Rochester, 55905 MN USA; Takeda Pharmaceuticals International, Inc., One Takeda Parkway, Deerfield, 60015 IL USA; University of Alabama, Faculty Office Tower 805B, 510 20th Street S, Birmingham, 35294 AL USA

Unfortunately, the original version of this article [1] contained an error. The Fig. 1 was included incorrectly. The correct Fig. 1 can be found below.

**Fig. 1:**
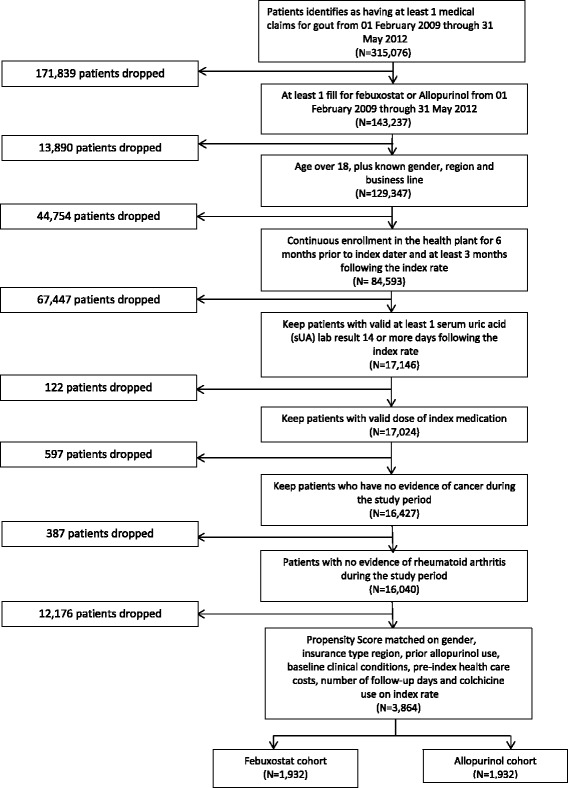
ᅟ
