# Supplementary material for: Comparative effectiveness of urate lowering with febuxostat versus allopurinol in gout: analyses from large U.S. managed care cohort
Source: Arthritis Res Ther. 2015 May 12;17(1):120. doi: 10.1186/s13075-015-0624-3 (PMC4427980; doi:10.1186/s13075-015-0624-3)
Supplement: Additional file 1: — Patient treatment patterns among full matched study population (N = 3,864). [file 13075_2015_624_MOESM1_ESM.docx]

**Additional file**

**Additional file 1. Patient treatment patterns among full matched study population (N=3,864)**

| **Post-index treatment patterns** | **Total (N=3,864)** | | **Febuxostat (N=1,932)** | | **Allopurinol (N=1,932)** | | **Febuxostat vs. Allopurinol p-value** |
| --- | --- | --- | --- | --- | --- | --- | --- |
|  | N | % | N | % | n | % |  |
| **Prophylaxis Treatment** | 2,076 | 53.7% | 1,164 | 60.3% | 912 | 47.2% |  |
| Steroids | 1,331 | 34.5% | 751 | 38.9% | 580 | 30.0% | <0.001 |
| NSAID | 780 | 20.2% | 426 | 22.1% | 354 | 18.3% | 0.004 |
| Colchicines | 805 | 20.8% | 513 | 26.6% | 292 | 15.1% | <0.001 |
| **Index Febuxostat Dose** |  |  |  |  |  |  |  |
| 40mg | 1,557 | 40.3% | 1,557 | 80.6% | - | - | - |
| 80mg | 375 | 9.7% | 375 | 19.4% | - | - | - |
| **Index Allopurinol Dose** |  |  |  |  |  |  |  |
| 100mg | 681 | 17.6% | - | - | 681 | 35.3% | - |
| 101-299mg | 278 | 7.2% | - | - | 278 | 14.4% | - |
| 300mg | 885 | 22.9% | - | - | 885 | 45.8% | - |
| >300mg | 88 | 2.3% | - | - | 88 | 4.6% | - |
| **Index Medication Dose Change** | 997 | 25.8% | 398 | 20.6% | 599 | 31.0% | <0.001 |
| **Percent of Dose Change** |  |  |  |  |  |  |  |
| 51-99% reduction | 59 | 1.5% | 0 | 0.0% | 59 | 3.1% | <0.001 |
| 1-50% reduction | 122 | 3.2% | 34 | 1.8% | 88 | 4.6% | <0.001 |
| 1-50% increase | 90 | 2.3% | 3 | 0.2% | 87 | 4.5% | <0.001 |
| 51-100% increase | 519 | 13.4% | 333 | 17.2% | 186 | 9.6% | <0.001 |
| >100% increase | 207 | 5.4% | 28 | 1.5% | 179 | 9.3% | <0.001 |
|  | **Mean** | **SD** | **Mean** | **SD** | **Mean** | **SD** |  |
| **Time to Dose Change (days)** | 235 | 243 | 237 | 218 | 233 | 259 | 0.043 |
| **Index medication dose immediately prior to sUA goal attainment of <6 mg/dl** | N/A | N/A | 54.1 | 22.0 | 279.0 | 112.7 | <0.001 |
| **Index medication dose immediately prior to sUA goal attainment of <5 mg/dl** | N/A | N/A | 55.5 | 23.9 | 306.5 | 129.3 | <0.001 |

N/A, not applicable, since the dose for febuxostat and allopurinol are different and averaging of doses is

Additional file 2. Post-index Clinical Characteristics in the propensity score matched cohorts (%)

| **Clinical Condition** | **Total** | **Febuxostat** | **Allopurinol** |
| --- | --- | --- | --- |
|  | **(N=3,864)** | **(N=1,932)** | **(N=1,932)** |
|  |  |  |  |
| **Kidney failure** | 2.3 | 3.2 | 1.4 |
| **Kidney stones** | 0.6 | 0.5 | 0.8 |
| **Dialysis** | 1.6 | 1.6 | 1.7 |
| **Angina** | 3.4 | 3.5 | 3.3 |
| **Diabetes** | 13.7 | 14.9 | 12.4 |
| **Coronary artery disease** | 13.3 | 13.2 | 13.4 |
| **Heart failure** | 5.0 | 5.6 | 4.3 |
| **Myocardial infarction** | 3.0 | 3.2 | 2.9 |
| **Stroke** | 5.5 | 6.0 | 5.0 |
| **Peripheral arterial disease** | 2.7 | 3.1 | 2.3 |
| **Osteoarthritis** | 25.2 | 26.9 | 23.6 |
| **Hypertension** | 40.4 | 39.4 | 41.5 |
| **Hyperlipidemia** | 54.2 | 54.9 | 53.6 |
| **Alcohol Abuse** | 1.4 | 1.3 | 1.5 |
