# Supplementary material for: Comparative effectiveness of urate lowering with febuxostat versus allopurinol in gout: analyses from large U.S. managed care cohort
Source: Arthritis Res Ther. 2015 May 12;17(1):120. doi: 10.1186/s13075-015-0624-3 (PMC4427980; doi:10.1186/s13075-015-0624-3)
Supplement: Additional file 2: — Post-index clinical characteristics in the propensity score-matched cohorts (%). [file 13075_2015_624_MOESM2_ESM.doc]

Additional file 2. Post-index Clinical Characteristics in the propensity score matched cohorts (%)

| **Clinical Condition** | **Total** | **Febuxostat** | **Allopurinol** |
| --- | --- | --- | --- |
|  | **(N=3,864)** | **(N=1,932)** | **(N=1,932)** |
|  |  |  |  |
| **Kidney failure** | 2.3 | 3.2 | 1.4 |
| **Kidney stones** | 0.6 | 0.5 | 0.8 |
| **Dialysis** | 1.6 | 1.6 | 1.7 |
| **Angina** | 3.4 | 3.5 | 3.3 |
| **Diabetes** | 13.7 | 14.9 | 12.4 |
| **Coronary artery disease** | 13.3 | 13.2 | 13.4 |
| **Heart failure** | 5.0 | 5.6 | 4.3 |
| **Myocardial infarction** | 3.0 | 3.2 | 2.9 |
| **Stroke** | 5.5 | 6.0 | 5.0 |
| **Peripheral arterial disease** | 2.7 | 3.1 | 2.3 |
| **Osteoarthritis** | 25.2 | 26.9 | 23.6 |
| **Hypertension** | 40.4 | 39.4 | 41.5 |
| **Hyperlipidemia** | 54.2 | 54.9 | 53.6 |
| **Alcohol Abuse** | 1.4 | 1.3 | 1.5 |
